# Clinician-reported Gloucester Comfort Scale scores underestimate patient discomfort and pain during colonoscopy: insights from comparison with a patient-reported experience measure

**DOI:** 10.1055/a-2528-5578

**Published:** 2025-02-28

**Authors:** Querijn N. E. van Bokhorst, Charmayne V. Geerlings, Manon van der Vlugt, Karlijn J. Nass, Jos W. Borkent, Laura J. Neilson, Paul Fockens, Colin J. Rees, Evelien Dekker

**Affiliations:** 1 Department of Gastroenterology and Hepatology, Amsterdam University Medical Center, Location VU Medical Center, Amsterdam, Netherlands; 2 571165 Amsterdam Gastroenterology Endocrinology Metabolism, Amsterdam, Netherlands; 3 Cancer Center Amsterdam, Amsterdam, Netherlands; 4 Department of Gastroenterology, Bergman Clinics, Amsterdam, Netherlands; 5 1322 Department of Internal Medicine, Rijnstate Hospital, Arnhem, Netherlands; 6 6032 Lectorate for Nutrition, Dietetics and Lifestyle, HAN University of Applied Sciences, Nijmegen, Netherlands; 7 7413 Department of Gastroenterology, South Tyneside and Sunderland NHS Foundation Trust, South Shields, United Kingdom of Great Britain and Northern Ireland; 8 151476 Faculty of Medical Sciences, Newcastle University Population Health Sciences Institute, Newcastle upon Tyne, United Kingdom of Great Britain and Northern Ireland

## Abstract

**Background:**

Patient experience is a fundamental element of colonoscopy. The Gloucester Comfort Scale (GCS) is used by clinicians to report patient comfort. However, insights regarding the extent to which clinician-reported GCS scores represent the patient’s experience are lacking. We assessed the level of agreement between clinician-reported GCS scores and patient-reported discomfort and pain.

**Methods:**

Consecutive patients undergoing colonoscopy at two Dutch endoscopy clinics were included. Patient comfort during colonoscopy was reported using the GCS (1–5 scale). Patients’ colonoscopy experiences were assessed using the Newcastle ENDOPREM, a validated endoscopy patient-reported experience measure (PREM). Patients reported both discomfort and pain levels experienced during colonoscopy on a 1–5 scale. Levels of agreement were assessed using Cohen’s kappa statistic.

**Results:**

For 243 included patients, the GCS score was higher than the PREM discomfort score in 52 patients (21%) and lower in 72 (30%). GCS score was higher than the PREM pain score in 39 patients (16%) and lower in 71 (29%). Moderate-to-severe discomfort and pain (scores ≥3) were reported by 53 patients (22%) for discomfort and 60 patients (25%) for pain. For these patients, the GCS underestimated discomfort and pain levels in almost all cases (discomfort 49/53 [92%], pain 54/60 [90%]). Agreement between GCS scores and PREM discomfort and pain scores were minimal (Cohen’s κ 0.34) and weak (Cohen’s κ 0.47), respectively.

**Conclusions:**

Clinician-reported GCS scores frequently underestimated the level of discomfort and pain reported by patients. For accurate monitoring of patients’ colonoscopy experiences, the use of PREMs should be considered.

## Graphical Abstract

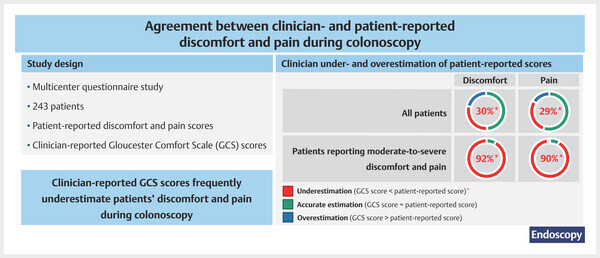

## Introduction

Colonoscopy is the preferred method for diagnosing colorectal diseases. However, patients may perceive colonoscopy as an uncomfortable, painful, and embarrassing procedure. A previous negative colonoscopy experience can lead to decreased patient satisfaction and a negative attitude toward colonoscopy, potentially hampering participation in future screening activities, adherence to surveillance recommendations, and diagnostic work-up of colorectal symptoms 1 2 3 4 .

Current quality guidelines for colonoscopy include patient experience as one of the key performance measures and recommend routine measurement of patient experience. However, these guidelines also acknowledge the lack of a standardized approach for this purpose 5 6 7 . Consequently, patient experience is currently mostly derived and measured by clinicians (nurses and endoscopists) using rating scales such as the Gloucester Comfort Scale (GCS) 8 . The GCS is designed to measure and report patient experience in terms of patient comfort during colonoscopy. Despite widespread use of the GCS, preliminary studies have suggested that clinician-reported GCS scores may not accurately reflect the colonoscopy experience from the patient’s perspective 9 10 . However, specific insights regarding the extent of discrepancy between clinician-reported GCS scores and patient-reported experience in terms of colonoscopy-related discomfort and pain are lacking.

Recently, a new patient-reported experience measure (PREM) for gastrointestinal endoscopy was developed and validated: the Newcastle ENDOPREM 11 12 . This questionnaire is designed to provide a comprehensive insight into the patient’s endoscopy experience, covering all aspects of the endoscopy procedure, from referral up until communication of test results and follow-up arrangements. The PREM also assesses patient experience in terms of the level of discomfort and pain during colonoscopy.

The comprehensive information provided by the Newcastle ENDOPREM can help to compare colonoscopy experience from the clinician and patient perspectives. In this study, we aimed to use this information to evaluate the extent of discrepancy between clinician-reported GCS scores and patient-reported levels of colonoscopy-related discomfort and pain. Moreover, we aimed to identify physical, procedural, and emotional factors associated with moderate-to-severe levels of discomfort and pain, and to identify patients in whom clinicians were more likely to over- or underestimate discomfort and pain using the GCS.

## Methods

### Study design

Consecutive patients undergoing colonoscopy at Bergman Clinics, Amsterdam (center A) and Amsterdam University Medical Center, Amsterdam (center B) were invited to complete the Newcastle ENDOPREM 11 12 . Agreement between clinician- and patient-reported colonoscopy experience was assessed using clinician-reported GCS scores and patient-reported levels of discomfort and pain. In addition, patient-specific and procedural characteristics, as well as patient-reported experiences regarding various aspects of the colonoscopy procedure, were used to identify factors that may be associated with greater colonoscopy-related discomfort and pain.

The Institutional Review Board of the Amsterdam University Medical Center, Amsterdam (2023.0266) decided that formal revision according to the Medical Research Involving Human Subjects Act (WMO) was not required.

### Patient recruitment and selection

Patients were recruited between July 2023 and February 2024. All adult patients (≥18 years) scheduled for outpatient colonoscopy for any indication and able to complete a questionnaire in Dutch (alone or with assistance) were considered eligible. Eligible patients were provided with a study pack, consisting of an invitation letter, participant information sheets (including a consent form), and questionnaire. Study packs were distributed by front office personnel at the endoscopy clinic on the day of the procedure, before the start of the colonoscopy. As such, endoscopists and nurses were unaware of the ongoing study. The questionnaire and signed consent form could be returned using a prepaid envelope.

To enable monitoring of the response rate, all questionnaires were numbered. Patients were identified based on returned questionnaires with the completed consent form. Patients were retrospectively excluded if their endoscopy report did not include a GCS score, if they underwent a colonoscopy under propofol sedation (i.e. unconscious or deep sedation 13 ), if they had a medical history of extensive colorectal surgery (e.g. subtotal colectomy), if they underwent a procedure other than intended complete colonoscopy (e.g. sigmoidoscopy), or if they did not report both a discomfort and pain score within the questionnaire.

To minimize the effect of recall bias, patients were requested to complete the questionnaire at home within 2 days (48 hours) of the colonoscopy. However, patients were permitted to complete the questionnaire within a 30-day period following their colonoscopy. Questionnaires completed beyond the 30-day window were excluded, as patients’ perceptions of their colonoscopy experience may change over a longer period after the procedure 14 .

### Colonoscopies

Colonoscopies were performed by both gastroenterologists and supervised gastroenterologists in training (0–4 years of endoscopy experience). While participating centers were affiliated, endoscopies at both centers were performed by the same rotating group of endoscopists. Both centers used the same sedation protocol. Prior to the procedure, all patients were asked whether they wished to receive a sedative (midazolam), an analgesic (fentanyl), or both. For patients willing to receive medication, doses of 2.5 mg midazolam and 0.05 mg fentanyl were considered standard. Administration of additional medication was at the discretion of the endoscopist considering the patient’s previous colonoscopy experience and peri-procedural comfort. The level of sedation was reported by the endoscopist using the Leeds Scale ( Table 1 ).

**Table:** **Table 1** Gloucester Comfort Scale for reporting patient comfort and Leeds Scale for reporting degree of sedation during colonoscopy.

Scale and score	Degree of discomfort/sedation	Corresponding PREM numeric rating scale scores ^1^
Gloucester Comfort Scale		
1	None to very mild – no discomfort, resting comfortably throughout	0–2
2	Mild – one or two episodes of mild discomfort, well tolerated	3–4
3	Moderate – more than two episodes of mild discomfort, adequately tolerated	5–6
4	Severe – significant discomfort, experienced several times during the procedure	7–8
5	Very severe – extreme discomfort, experienced frequently during the test	9–10
Leeds Scale		
1	Fully awake	N/A
2	Sleepy/drowsy	N/A
3	Sleeps, responds to voice	N/A
4	Sleeps, responds to touch	N/A
5	Unresponsive	N/A
N/A, not applicable; PREM, patient-reported experience measure. ^1^ To allow for study analyses, patient-reported discomfort and pain scores as reported on a 0–10 scale within the Newcastle ENDOPREM questionnaire were converted to a 1–5 scale.		

Patient comfort during colonoscopy was assessed using the GCS ( Table 1 ) 8 . As part of routine practice, at center A the GCS scores were reported by the attending endoscopy nurse, while at center B the GCS scores were reported by the endoscopist. In this study, clinician-reported GCS scores refer to the combined scores recorded by both nurses and endoscopists. Scores reported solely by nurses or endoscopists will be referred to as nurse-reported and endoscopist-reported scores, respectively. Considering the “textbook process” composite quality measure for colonoscopy, high GCS scores were defined as scores ≥3 15 . Overestimation was defined as a GCS score higher than the patient-reported score, while underestimation was defined as a GCS score lower than the patient-reported score.

### Questionnaire

The Newcastle ENDOPREM is a comprehensive PREM for gastrointestinal endoscopy. Development and validation of this PREM have been described elsewhere 11 12 . The questionnaire comprises seven sections (labeled A to G) and is structured to follow the temporal phases of endoscopic procedures. Section A enquires about general patient and procedure information. Subsequent sections (B to F) enquire about the patient’s experience before coming to the hospital (e.g. referral and patient’s expectations), when preparing for the procedure at home (i.e. bowel preparation), when arriving at the hospital (e.g. privacy while waiting for the procedure), during the procedure, and after the procedure, respectively. Section G enquires about the patient’s overall experience. More detailed insights into the composition and aims of each section have been previously described 11 12 16 .

Most questions ask patients to indicate the extent to which they agree with specific statements on a 5-point Likert scale ranging from “strongly agree” to “strongly disagree.” The questionnaire enquires about the levels of discomfort and pain as experienced during the procedure using a 0–10 numeric rating scale, with a score of 0 representing no discomfort or pain and 10 representing the worst discomfort or pain imaginable. To compare patient-reported scores with GCS scores using similar scales, the scores as reported on the 11-point numeric scales were converted to 5-point scales ( Table 1 ). Moderate-to-severe patient-reported discomfort and pain were defined as scores ≥3 on the 5-point scales.

For the purposes of this study, the original Newcastle ENDOPREM questionnaire was modified for colonoscopy, translated to Dutch, and contextualized for the Dutch population (see **Appendix 1s** in the online-only Supplementary material). The process of development and validation of this adapted version of the PREM will be described elsewhere.

### Statistical analysis

Patient and procedure characteristics were described using descriptive statistics. Normality of data was checked using stem-and-leaf and QQ-plots. Levels of agreement between clinician-reported GCS scores and converted patient-reported discomfort and pain scores were assessed using the Cohen’s kappa statistic with squared weights 17 . Exploratory post hoc analyses were performed to compare levels of agreement for different types of assessors (nurses and endoscopists), endoscopists with different levels of endoscopy experience, and for patients with different degrees of sedation. The strength and direction of the association between patient-reported discomfort and pain scores were examined using the Goodman and Kruskal’s gamma statistic 18 . The 95%CIs around the reported Cohen’s kappa and Goodman and Kruskal’s gamma values were calculated using bootstrapping with 1000 iterations.

Logistic regressions were used to assess the putative association between moderate-to-severe patient-reported discomfort and pain (dependent variables) and various patient- and procedure-related factors (independent variables). We adjusted for age (dichotomized at 55 years) and sex, as these factors are already well known to increase the likelihood of moderate-to-severe discomfort and pain during colonoscopy 19 20 21 22 . We adjusted for endoscopy center to account for potential variations across locations. Additional univariable regression analyses were performed using over- and underestimation of discomfort and pain as dependent variables, as well as analyses using experience-related predictor variables as independent variables (an overview of questions corresponding to each of the experience-related and emotional domains is shown in **Table 1s** ). Results of the regression analyses were reported as odds ratios (ORs) with 95%CIs.

All analyses were performed using R version 4.2.1 (R Foundation for Statistical Computing, Vienna, Austria). Two-sided *P* values <0.05 were considered statistically significant.

### Sample size

We based our sample size on the study aim to identify patient- and procedure-related factors associated with moderate-to-severe patient-reported discomfort and pain. We aimed for our data to allow for multivariable regression analyses with 5 degrees of freedom. In order to not cross the “10 events per variable” criterion 23 , this required inclusion of at least 50 patients with moderate-to-high scores for both discomfort and pain. Assuming an incidence of moderate-to-high pain scores of about 21% 24 , an estimated number of 238 participants was required. Accounting for an estimated questionnaire response rate of 48% 12 and a 10% exclusion rate, we estimated that distribution of approximately 545 questionnaires was needed.

## Results

**Fig. 1:**
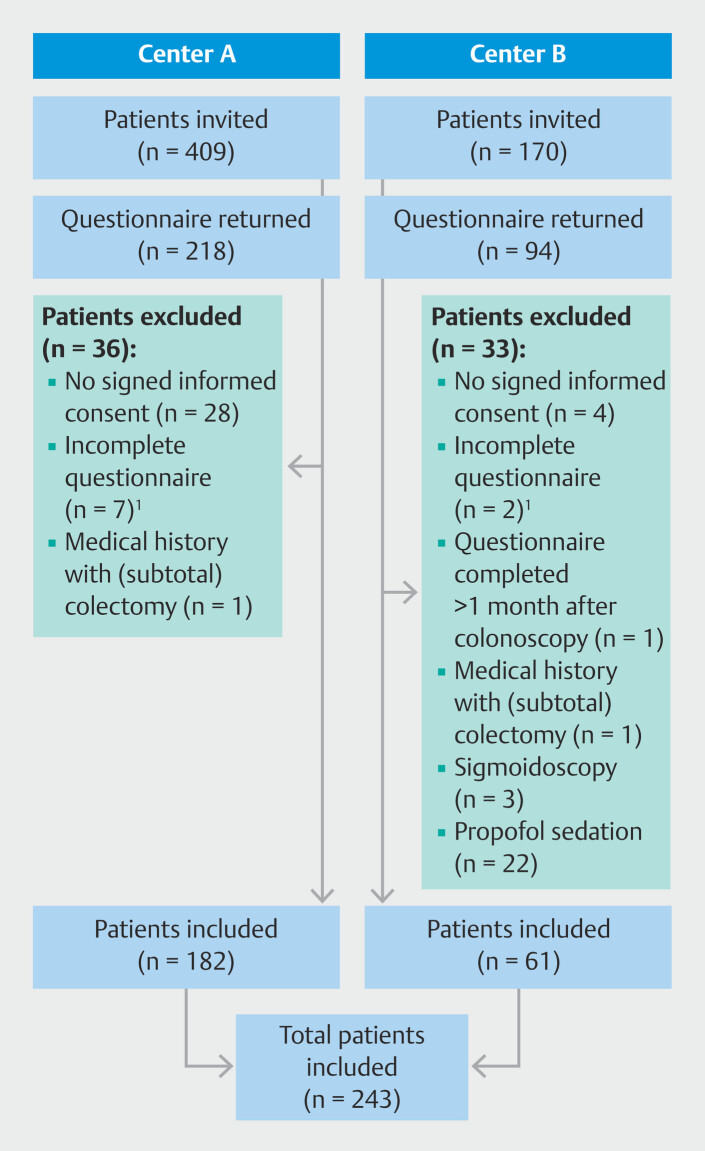
Study flow chart: patient invitation and inclusion process. ^1^ Questionnaires were considered incomplete if they did not report both discomfort and pain scores.

A total of 579 patients were invited, of whom 312 (54%) returned the questionnaire. Due to the staggered return of questionnaires, the number of invitees slightly surpassed the estimated required number. A total of 69 respondents (22%) were excluded, resulting in a total of 243 included patients. Reasons for exclusions are reported in Fig. 1 . Most questionnaires were completed within the first 48 hours (198/243, 81%) or the first week (225/243, 93%) after colonoscopy.

Patient and procedure characteristics are shown in Table 2 and Table 3 . The majority of patients were female (51%) and the median age was 65 (interquartile range 58–71) years. Patients underwent colonoscopy for a surveillance indication (after previously detected polyps or colorectal cancer or due to an increased familial risk of colorectal cancer; 40%), after a positive fecal immunochemical test within the context of the national colorectal cancer screening program (CRCSP; 34%), for symptoms (24%), or for other indications (2%) ( Table 3 , **Table 2s** ). Colonoscopies were performed under conscious sedation with midazolam and/or fentanyl in 215 patients (88%), while 28 patients (12%) received no medication.

**Table:** **Table 2** Characteristics of included patients.

	Center A (n = 182)	Center B (n = 61)	All (n = 243)
Sex, n (%)			
Male	88 (48)	30 (49)	118 (49)
Female	94 (52)	31 (51)	125 (51)
Age, median (IQR), years	65 (59–72)	61 (48–66)	65 (58–71)
BMI, median (IQR), kg/m2	25 (23–28)	25 (23–29)	25 (23–29)
Educational level ^1^ , n (%)			
Low	43 (24)	9 (15)	52 (21)
Intermediate or high	129 (71)	51 (84)	180 (74)
Not available	10 (6)	1 (2)	11 (5)
ASA score, n (%)			
ASA I	73 (40)	15 (25)	88 (36)
ASA II	108 (59)	46 (75)	154 (64)
ASA III	1 (<1)	0 (0)	1 (<1)
Previous colonoscopy, n (%)			
No	104 (57)	8 (13)	112 (46)
Yes	78 (43)	53 (87)	131 (54)
Previous abdominal surgery ^2^ , n (%)			
No	138 (76)	46 (75)	184 (76)
Yes	44 (24)	15 (25)	59 (24)
Diverticulosis ^3^ , n (%)			
No	82 (45)	50 (82)	132 (54)
Yes	100 (55)	11 (18)	111 (46)
ASA, American Association of Anesthesiologists; BMI, body mass index; IQR, interquartile range. ^1^ Education level according to ISCED-11. Patients were considered to have an intermediate or high educational level if they had at least an upper secondary or university degree. ^2^ Defined as any (laparoscopic) surgical procedure in which the abdominal cavity was entered, excluding diagnostic laparoscopies and cesarean sections. ^3^ Of all patients with diverticulosis, 103/111 (93%) had diverticulosis within the sigmoid colon; 10 patients had a medical history reporting at least 1 episode of diverticulitis; 10 patients had diverticulosis with stricture(s), 6 of whom had passage problems.			

**Table:** **Table 3** Characteristics of colonoscopies.

	Center A (n = 182)	Center B (n = 61)	All (n = 243)
Indication ^1^			
CRCSP	82 (45)	0 (0)	82 (34)
Surveillance ^2^	48 (26)	50 (82)	98 (40)
Symptoms and other	52 (29)	11 (18)	63 (26)
Quality of bowel preparation ^3^			
Excellent	159 (89)	48 (79)	207 (85)
Sufficient	19 (10)	6 (10)	25 (10)
Not available	4 (2)	7 (12)	11 (4.5)
Cecal intubation			
No	6 (3)	2 (3)	8 (3)
Yes	176 (97)	59 (97)	235 (97)
Endoscopist ^4^			
Gastroenterologist (CRCSP accredited)	124 (68)	24 (39)	148 (61)
Gastroenterologist (not CRCSP accredited)	26 (14)	8 (13)	34 (14)
Gastroenterologist in training	32 (18)	29 (48)	61 (25)
Analgesic and sedative medication			
Midazolam and fentanyl	149 (82)	56 (92)	205 (84)
Midazolam only	2 (1)	0 (0)	2 (<1)
Fentanyl only	6 (3)	2 (3)	8 (3)
None	25 (14)	3 (5)	28 (12)
Leeds score			
1	95 (52)	21 (34)	116 (48)
2	68 (37)	32 (52)	100 (41)
≥3	17 (9)	8 (13)	25 (10)
Not reported	2 (1)	0 (0)	2 (<1)
CRCSP, colorectal cancer screening Program. ^1^ An overview of colonoscopy indications belonging to each category is shown in **Table 2s** . ^2^ Surveillance colonoscopies after previously detected polyps or colorectal cancer and surveillance colonoscopies in individuals with an increased familial risk for colorectal cancer. ^3^ Based on Boston Bowel Preparation Scale score: poor (<6 points), sufficient (6–8 points), excellent (9 points). ^4^ To assure colonoscopy quality for colonoscopies performed within the context of the Dutch CRCSP, all endoscopists performing these procedures have to be accredited. The endoscopist accreditation program consists of three modules: (1) colonoscopy registration module, (2) theoretical e-learning module combined with online assessment of the acquired knowledge, and (3) a practical evaluation of colonoscopy and polypectomy skills.			

### Agreement between clinician-reported GCS scores and patient-reported scores

**Fig. 2:**
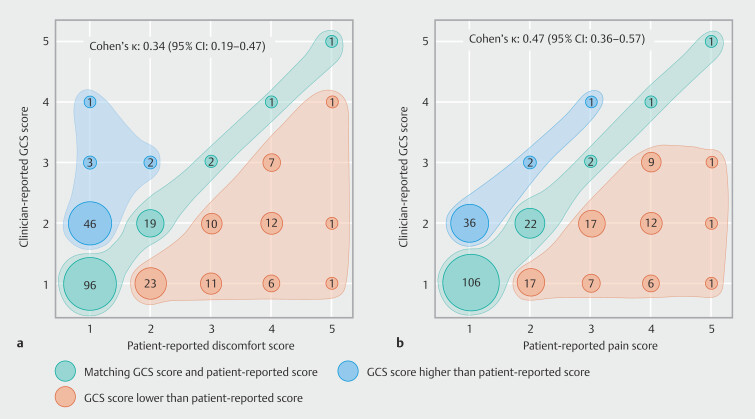
Plots illustrating the level of agreement between clinician-reported Gloucester Comfort Scale (GCS) scores and patient-reported scores. **a** Discomfort scores. **b** Pain scores. The number of patients that is represented by each data point within the plots is indicated by the size of each data point and the number within each data point. The Cohen’s kappa statistic reports the level of agreement between clinician- and patient-reported scores, and indicates that the level of agreement between GCS scores and patient-reported scores is minimal for discomfort (Cohen’s κ 0.34 [95%CI 0.19–0.47]) and weak for pain (Cohen’s κ 0.47 [95%CI 0.34–0.57]).

GCS scores matched patient-reported discomfort scores in 119 patients (49%), were lower in 72 patients (30%), and were higher in 52 patients (21%) ( Fig. 2 **a** ). GCS scores matched patient-reported pain scores in 133 patients (55%), were lower in 71 patients (29%), and were higher in 39 patients (16%) ( Fig. 2 **b** ). A GCS score ≥3 was reported for 18 patients (7%). Moderate-to-severe discomfort and pain were reported by 53 (22%) and 60 (25%) patients, respectively. Among these patients, the GCS score was lower than the patient-reported score for discomfort in 49/53 (92%) and for pain in 54/60 (90%).

Agreement between the GCS and patient-reported scores was minimal for discomfort (Cohen’s κ 0.34 [95%CI 0.19–0.47]) and weak for pain (Cohen’s κ 0.47 [95%CI 0.34–0.57]) 17 . The Goodman and Kruskal’s gamma statistic showed a strong positive association between patient-reported discomfort and pain scores (Goodman and Kruskal’s γ 0.78 [95%CI 0.69–0.86]) ( **Fig. 1s** ) 18 . Exploratory analyses revealed Cohen’s κ values of 0.34 (95%CI 0.15–0.49) and 0.52 (95%CI 0.38–0.65) for the level of agreement between nurse-reported scores and patient-reported discomfort and pain scores, respectively. The corresponding Cohen’s κ values for endoscopist-reported scores were 0.32 (95%CI 0.10–0.54) and 0.33 (95%CI 0.15–0.52). Analyses only involving gastroenterologists in training showed Cohenʼs κ values of 0.47 (95%CI 0.25–0.64) for discomfort and 0.61 (95%CI 0.43–0.75) for pain, with corresponding values of 0.27 (95%CI 0.11–0.44) and 0.40 (95%CI 0.26–0.53) for analyses only involving certified gastroenterologists ( **Table 3s** ) 17 . Rates of moderate-to-severe discomfort and pain were comparable for patients completing the questionnaire within vs. beyond the allotted 2-day time frame (discomfort 44/198 [22%] vs. 9/45 [20%]; pain 48/198 [24%] vs. 12/45 [27%]).

### Sedation and discomfort and pain scores

An overview of reported GCS and discomfort and pain scores in relation to the (dose of) administered medication is shown in **Table 4s** . All patients with a GCS ≥3 (n = 18) had received medication, of whom 15/18 (83%) had received higher than standard doses. Of the patients who reported moderate-to-severe discomfort or pain, 48/53 (91%) and 55/60 (92%) patients, respectively, received medication. Medication was at a higher than standard dose for 19/53 patients (36%) reporting moderate-to-severe discomfort and for 27/60 patients (45%) reporting moderate-to-severe pain.

For patients with a Leeds score of 1 (n = 116), the Cohen’s κ values for agreement between GCS scores and patient-reported discomfort and pain scores were 0.20 (95%CI 0.01–0.37) and 0.38 (95%CI 0.21–0.55), respectively. The corresponding Cohen’s κ values for patients with a Leeds score of ≥2 (n = 125) were 0.33 (95%CI 0.15–0.50) and 0.45 (95%CI 0.28–0.59) ( **Table 3s** ) 17 . The strength of the association between discomfort and pain scores was similar in both groups (Goodman and Kruskal’s γ values of 0.74 [95%CI 0.55–0.87] and 0.79 [95%CI 0.67–0.88]) 18 .

### Factors associated with moderate-to-severe patient-reported scores

Multivariable regression analyses revealed a significant association between female sex and both moderate-to-severe discomfort and pain. Age <55 years was associated with moderate-to-severe discomfort, while diverticulosis of the sigmoid showed a significant association with moderate-to-severe pain. Finally, the likelihood of moderate-to-severe discomfort and pain was significantly lower for CRCSP colonoscopies compared with colonoscopies for other indications ( Table 4 , Table 5 , **Table 5s** , **Table 6s** ).

**Table:** **Table 4** Univariable and multivariable regression analyses assessing the association between different patient- and procedural factors and moderate-to-severe patient-reported discomfort.

	Univariable analysis		Multivariable analysis ^1^	
	OR (95%CI)	*P* value	OR (95%CI)	*P* value
Sex (vs. male)				
Female	2.68 (1.40–5.15)	0.003	2.46 (1.25–4.86)	0.009
Age (vs. ≥55 years)				
<55 years	4.14 (2.00–8.56)	<0.001	2.91 (1.33–6.36)	0.007
Educational level ^2^ (vs. low)				
Medium or high	1.45 (0.66–3.23)	0.36	1.12 (0.48–2.59)	0.80
BMI ^3^ (vs. 18.5–25.0)				
>25.0	0.74 (0.40–1.36)	0.33	0.98 (0.51–1.90)	0.96
Previous abdominal surgery (vs. none)				
Yes	1.48 (0.75–2.91)	0.26	1.40 (0.68–2.89)	0.36
Previous colonoscopy (vs. none)				
Yes	1.55 (0.83–2.89)	0.17	1.16 (0.57–2.37)	0.68
Diverticulosis sigmoid (vs. none)				
Yes	0.86 (0.46–1.61)	0.64	1.64 (0.79–3.41)	0.19
Colonoscopy indication (vs. CRCSP)				
Surveillance and familial risk	5.07 (1.98–12.97)	<0.001	3.22 (1.13–9.21)	0.03
Symptoms and other	5.47 (2.03–14.72)	<0.001	3.77 (1.33–10.70)	0.01
Endoscopist experience (vs. gastroenterologist)				
Gastroenterologist in training	1.97 (1.02–3.80)	0.04	1.60 (0.77–3.32)	0.16
Endoscopist type ^4^ (vs. CRCSP accredited				
Not CRCSP accredited	0.52 (0.17–1.60)	0.26	0.41 (0.12–1.40)	0.16
BMI, body mass index; CRCSP, colorectal cancer screening program; OR, odds ratio. ^1^ Adjusted for sex, age (dichotomized at 55 years), and endoscopy center. ^2^ Education level according to ISCED-11. Patients were considered to have an intermediate or high educational level if they had at least an upper secondary or university degree. ^3^ One patient with BMI <18.5 was excluded from the analyses. ^4^ To assure colonoscopy quality for colonoscopies performed within the context of the Dutch CRCSP, all endoscopists performing these procedures have to be accredited. The endoscopist accreditation program consists of three modules: (1) colonoscopy registration module, (2) theoretical e-learning module combined with online assessment of the acquired knowledge, and (3) a practical evaluation of colonoscopy and polypectomy skills.				

**Table:** **Table 5** Univariable and multivariable regression analyses assessing the association between different patient- and procedural factors and moderate-to-severe patient-reported pain.

	Univariable analysis		Multivariable analysis ^1^	
	OR (95%CI)	*P* value	OR (95%CI)	*P* value
Sex (vs. male)				
Female	2.81 (1.51–5.23)	<0.001	2.68 (1.41–5.09)	0.003
Age (vs. ≥55 years)				
<55 years	2.89 (1.41–5.92)	0.004	2.01 (0.92–4.36)	0.08
Educational level ^2^ (vs. low)				
Medium or high	1.18 (0.57–2.43)	0.66	0.99 (0.46–2.12)	0.97
BMI ^3^ (vs. 18.5–25.0)				
>25.0	0.71 (0.40–1.27)	0.25	0.86 (0.46–1.60)	0.63
Previous abdominal surgery (vs. none)				
Yes	1.48 (0.77–2.85)	0.24	1.37 (0.69–2.72)	0.37
Previous colonoscopy (vs. none)				
Yes	1.16 (0.64–2.09)	0.62	0.85 (0.43–1.67)	0.64
Diverticulosis sigmoid (vs. none)				
Yes	1.38 (0.77–2.48)	0.25	2.26 (1.11–4.58)	0.02
Colonoscopy indication (vs. screening)				
Surveillance and familial risk	1.92 (0.31–4.03)	0.09	1.06 (0.43–2.61)	0.89
Symptoms and other	2.65 (1.20–5.85)	0.02	1.82 (0.78–4.29)	0.17
Endoscopist experience (vs. gastroenterologist)				
Gastroenterologist in training	1.40 (0.73–2.67)	0.32	1.10 (0.54–2.26)	0.79
Endoscopist type ^4^ (vs. CRCSP accredited				
Not CRCSP accredited	1.15 (0.64–2.09)	0.64	0.81 (0.31–2.11)	0.66
BMI, body mass index; CRCSP, colorectal cancer screening program; OR, odds ratio. ^1^ Adjusted for sex, age (dichotomized at 55 years) and endoscopy center. ^2^ Education level according to ISCED-11. Patients were considered to have an intermediate or high educational level if they had at least an upper secondary or university degree. ^3^ One patient with BMI <18.5 was excluded from the analyses. ^4^ To assure colonoscopy quality for colonoscopies performed within the context of the Dutch CRCSP, all endoscopists performing these procedures have to be accredited. The endoscopist accreditation program consists of three modules: (1) colonoscopy registration module, (2) theoretical e-learning module combined with online assessment of the acquired knowledge, and (3) a practical evaluation of colonoscopy and polypectomy skills.				

Univariable regression analyses between experience-related and emotional factors and patient-reported discomfort and pain levels showed that pre-procedural anxiety for both the procedure itself and procedure-related discomfort or pain, a bad experience with bowel preparation, a low sense of general comfort or support (from the medical staff), feelings of embarrassment, and a procedure duration longer than expected were significantly associated with both moderate-to-severe discomfort and pain. Additionally, unsatisfactory waiting times were associated with moderate-to-severe discomfort, while anxiety for the procedure results was associated with moderate-to-severe pain ( Table 6 , Table 7 ).

**Table:** **Table 6** Univariable regression analyses assessing the association between specific experience-related and emotional factors and moderate-to-severe patient-reported discomfort.

Domain	Disagree ^1^		Agree ^1^			
	No to mild, n (%)	Moderate to severe, n (%)	No to mild, n (%)	Moderate to severe, n (%)	OR (95%CI)	*P* value
Inadequate information	179 (80)	46 (20)	11 (61)	7 (39)	2.48 (0.91–6.74)	0.09
Anxiety: procedure in general	151 (79)	41 (21)	33 (73)	12 (27)	1.34 (0.64–2.82)	0.45
Anxiety: procedure results	157 (80)	39 (20)	27 (66)	14 (34)	1.65 (0.89–3.06)	0.11
Anxiety: procedure-related discomfort	111 (90)	13 (10)	73 (65)	40 (35)	4.68 (2.34–9.35)	<0.001
Anxiety: procedure-related pain	112 (91)	11 (9)	73 (63)	42 (37)	5.86 (2.83–12.11)	<0.001
Bad experience bowel preparation	73 (89)	9 (11)	115 (72)	44 (28)	3.10 (1.43–6.73)	0.002
Unsatisfactory waiting times	181 (80)	46 (20)	9 (56)	7 (44)	3.06 (1.08–8.65)	0.04
Insufficient privacy or unrespected dignity	179 (78)	50 (22)	11 (72)	3 (28)	0.98 (0.26–3.64)	0.97
Endoscopist with nonpreferred sex ^2^	185 (79)	50 (21)	4 (57)	3 (43)	2.77 (0.60–12.81)	0.21
Low sense of comfort and support (from the medical staff)	188 (81)	44 (19)	2 (18)	9 (82)	19.23 (4.01–92.14)	<0.001
Feelings of embarrassment	186 (80)	46 (20)	4 (36)	7 (64)	7.08 (1.99–25.20)	0.002
Longer procedure duration than expected	182 (80)	45 (20)	8 (50)	8 (50)	4.04 (1.44–11.36)	0.01
OR, odds ratio; CI, confidence interval. ^1^ Questions and criteria used for assigning patients to either the “disagree” or “agree” group are displayed in **Table 1s** . Patients who completed none of the questions related to each domain were excluded from the analyses. ^2^ Patients were assigned to the “disagree” group when the patient-preferred endoscopist sex matched the sex of the endoscopist who performed the procedure, or patients indicated not to have a preference regarding the endoscopist’s sex. Patients were assigned to the “agree” group when the patient-preferred endoscopist sex did not match the sex of the endoscopist who performed the procedure.						

**Table:** **Table 7** Univariable regression analyses assessing the association between specific experience-related and emotional factors and moderate-to-severe patient-reported pain.

Domain	Disagree ^1^		Agree ^1^			
	No to mild, n (%)	Moderate to severe, n (%)	No to mild, n (%)	Moderate to severe, n (%)	OR (95%CI)	*P* value
Inadequate information	170 (76)	55 (24)	13 (72)	5 (28)	1.19 (0.41–3.48)	0.76
Anxiety: procedure in general	147 (77)	45 (23)	30 (67)	15 (33)	1.63 (0.81–3.30)	0.18
Anxiety: procedure results	111 (80)	28 (20)	66 (67)	32 (33)	1.92 (1.06–3.47)	0.03
Anxiety: procedure-related discomfort	105 (85)	19 (15)	72 (64)	41 (36)	3.15 (1.69–5.86)	<0.001
Anxiety: procedure-related pain	108 (88)	15 (12)	70 (61)	45 (39)	4.63 (2.40–8.93)	<0.001
Bad experience bowel preparation	71 (87)	11 (13)	111 (70)	48 (30)	2.79 (1.36–5.73)	0.003
Unsatisfactory waiting times	173 (76)	54 (24)	10 (63)	6 (37)	1.92 (0.67–5.53)	0.23
Insufficient privacy or unrespected dignity	172 (75)	57 (25)	11 (79)	3 (21)	0.82 (0.22–3.05)	0.77
Endoscopist with unpreferred sex ^2^	179 (76)	56 (24)	3 (43)	4 (57)	4.26 (0.93–19.62)	0.06
Low sense of comfort and support (from the medical staff)	179 (77)	53 (23)	4 (36)	7 (64)	5.91 (1.67–20.96)	0.005
Feelings of embarrassment	178 (77)	54 (23)	5 (45)	6 (55)	3.96 (1.16–13.47)	0.03
Longer procedure duration than expected	178 (78)	49 (22)	5 (31)	11 (69)	7.99 (2.65–24.09)	<0.001
OR, odds ratio; CI, confidence interval. ^1^ Questions and criteria used for assigning patients to either the “disagree” or “agree” group are displayed in **Table 1s** . Patients that completed none of the questions related to each domain were excluded from the analyses. ^2^ Patients were assigned to the “disagree” group when the patient-preferred endoscopist sex matched the sex of the endoscopist who performed the procedure, or patients indicated not to have a preference regarding the endoscopist’s sex. Patients were assigned to the “agree” group when the patient-preferred endoscopist sex did not match the sex of the endoscopist who performed the procedure.						

### Factors associated with over- and underestimation of patient discomfort and pain using the GCS

We identified no factors significantly associated with overestimation of discomfort using the GCS. Female sex, age <55 years, a previous colonoscopy, and a colonoscopy with an indication other than for the CRCSP were significantly associated with underestimation of patient discomfort ( **Table 7s** ). Regarding pain, a significant association with both over- and underestimation was found for female sex, age <55 years, and a colonoscopy indication other than for the CRCSP ( **Table 8s** ).

## Discussion

This prospective questionnaire study is the first study to compare clinician-reported GCS scores with patient-reported colonoscopy-related discomfort and pain. We demonstrated that clinician-reported GCS scores poorly reflect the colonoscopy experience of the patient. In particular, for patients reporting moderate-to-severe levels of discomfort and pain, nurse- or endoscopist-reported GCS scores underestimated patient discomfort and pain in almost all cases.

The results of this study add to the existing evidence that clinician-derived assessments often do not match the patient-reported level of procedural pain, discomfort, or procedure tolerability 9 10 24 25 26 27 . Moreover, our study findings align with the results of previous studies demonstrating a moderate correlation between GCS scores and patient-reported pain scores, as well as significant underestimation of patient-reported procedure tolerability using the GCS 9 10 . The poor level of agreement between clinicians’ and patients’ perspectives, as reflected in Cohen’s κ values of 0.34 (95%CI 0.19–0.47) and 0.47 (95%CI 0.34–0.57), pertains to both the overestimation (up to 21%) and underestimation (up to 30%) of patient-reported discomfort and pain. However, from the clinical standpoint, underestimation seems of greater concern as it is more prevalent and is associated with potentially preventable negative colonoscopy experiences.

Underestimation of patient discomfort and pain might relate to several factors. Primarily, clinicians may tend to base their judgment of patient comfort on procedural difficulty, rather than on patient feedback 28 . The fact that endoscopists are likely to be primarily focused on (successful completion of) the procedure rather than the patient’s comfort might also explain why nurse-derived assessments more frequently aligned with patients’ experiences in our study, similarly to the findings of preliminary studies 24 25 27 . Moreover, there may be differences in the understanding of what constitutes tolerable discomfort and pain between clinicians and patients, while clinicians may also be less cautious in detecting signals of discomfort and pain in patients who lack classical risk factors (e.g. younger age) for an uncomfortable colonoscopy 27 . Our study also showed that discomfort and pain were often perceived as separate aspects of the colonoscopy: the discomfort and pain scores of 82/243 patients (34%) did not match. Colonoscopy-related pain seems mainly a physical phenomenon that is generally caused by bowel insufflation, traction, and looping of the endoscope during insertion. We identified younger age and sigmoid diverticulosis as factors associated with an increased likelihood of a painful colonoscopy, which corroborates earlier studies 19 20 21 22 . Optimizing medication regimens, choosing the endoscope that best suits the patient’s situation, and using add-on techniques such as magnetic endoscopic imaging might aid in reducing colonoscopy-related pain 29 30 .

In contrast to pain, discomfort may be more multifactorial and related to both the physical and emotional burden of the colonoscopy. This study illustrated that patients may be more likely to experience higher levels of discomfort when they experience anxiety, a low sense of general comfort or support (from the medical staff), or feelings of embarrassment. Moreover, factors such as a negative experience with bowel preparation, unsatisfactory waiting times, and a longer procedure duration than expected seem more likely to influence the level of discomfort than the level of pain. However, as aforementioned factors were mostly not only significantly associated with discomfort but also with pain, this implies that discomfort and pain are often intertwined. This is in line with the findings from preliminary studies illustrating that emotional burdens may lead to decreased acceptability of colonoscopy procedures and a higher incidence of painful colonoscopies 19 31 32 . Therefore, enquiring about and addressing emotional burdens of a colonoscopy might be of equal importance to the colonoscopy’s technical aspects when it comes to optimizing patients’ colonoscopy comfort. In doing so, clinicians should also be aware that emotional burdens regarding colonoscopy may be more prevalent in females 16 33 34 . This is emphasized by the significant association between female sex and moderate-to-severe discomfort in this study.

Adequate estimation of the patient’s level of discomfort and pain during colonoscopy is an essential step in initiating measures to improve patient comfort. In our study, 83% of patients with a GCS score ≥3 received more than the standard dose of sedative and/or analgesic medication. For patients reporting moderate-to-severe discomfort and pain, these percentages were considerably lower (36% and 43%, respectively). These numbers illustrate that recognition of moderate-to-severe patient discomfort or pain by clinicians generally leads to administration of additional medication. Therefore, the notably low percentage of patients who received additional medication while reporting moderate-to-severe discomfort or pain appears to be primarily a result of clinicians’ underestimation of patients’ levels of discomfort and pain. If patients’ discomfort and pain were more adequately appraised, these patients could likely have benefitted from additional medication. However, a preliminary study showed that an individual endoscopist’s medication practice and the comfort of their patients are not always directly related 35 , and therefore, enhancing the endoscopist’s overall colonoscopy practice (e.g. insertion technique, addressing emotional factors) might be at least as important as the endoscopist’s medication practice in optimizing patients’ experiences.

While accurate assessment of the level of discomfort and pain has proven to be difficult, awareness of factors that increase the likelihood of an uncomfortable or painful colonoscopy might aid clinicians in taking appropriate measures to improve patient’s colonoscopy experiences. As shown in this study, clinicians should be aware that for females, younger patients (i.e. <55 years), and patients undergoing colonoscopy outside the context of the CRCSP, underestimation of pain and discomfort is more common. In addition, a previous colonoscopy was identified as a risk factor for underestimation of moderate-to-severe pain. Finally, factors such as anxiety, embarrassment, and a low sense of general comfort and support (from the medical staff) should be appropriately addressed.

Clinicians should also be aware that the patient’s experience may be influenced by the patient’s pre-procedural expectations. For instance, patients anticipating a completely pain-free procedure might be more likely to report higher levels of discomfort and pain, as any discomfort and pain experienced will be unexpected. If patients are aware that procedure-related discomfort or pain is sometimes inevitable to accomplish a high-quality and complete colonoscopy, this might assure better (emotional) patient preparation and acceptance. The same applies to procedure duration: patients should know that procedure duration is dependent on the technical procedural difficulty and procedural findings, and can therefore be longer than expected. The beneficial effects of adequate patient information on patients’ colonoscopy experiences have been previously illustrated 14 32 . The current study supports these results, as the likelihood of experiencing moderate-to-severe discomfort and pain was lower for CRCSP colonoscopies compared with other indications. For CRCSP colonoscopies, the pre-procedural consultation involves a 30-minute face-to-face consultation, whereas for other indications the pre-procedural consultations are generally considerably shorter and conducted via telephone.

In the future, the use of PREMs in daily practice could help to reduce the considerable underestimation of patient discomfort and pain that is currently observed using the GCS. Moreover, PREMs could aid clinicians in identifying factors that increase the likelihood of an uncomfortable or painful colonoscopy. One of the main issues with incorporating a PREM into daily practice is that the distribution and processing of comprehensive PREMs can be time consuming and logistically challenging. Nevertheless, PREMs comprising only a few key questions could already provide useful information for improving (future) colonoscopy procedures for individual patients. Development and validation of a shortened PREM that still encompasses the full breadth of patient experience, and its implementation in routine practice, should therefore be considered a focus for future research. Facilitating completion of PREMs via online healthcare platforms may facilitate distribution and completion of PREMs without significant increases in workload for healthcare professionals 36 .

This study has several strengths. Primarily, this is the first study to specifically address discrepancies between clinician-reported GCS scores and patient-reported levels of both discomfort and pain related to colonoscopy. Moreover, a validated PREM was used to enquire about patient experience, patients undergoing colonoscopy for a wide variety of indications were included, and clinicians were unaware of the ongoing study (i.e. distribution of the PREM). Therefore, this study provides realistic insights into daily practice. Furthermore, while patients were asked to complete the PREM after discharge from the endoscopy ward, effects of sedation were likely to have worn off.

One of the study’s limitations concerns the sample size. Our study was not primarily powered for multivariable regression analyses regarding over- and underestimation of discomfort and pain, as well as analyses involving the questionnaire-derived experience-related and emotional factors. As such, the reported findings warrant further exploration on a larger scale to allow for adequate adjustment for potential confounding factors. Moreover, the exploratory post hoc analyses were performed within smaller subgroups of our study population; therefore, results of these analyses should be interpreted with some caution. Notwithstanding, as these analyses suggest that levels of agreement may differ between nurses and endoscopists, between endoscopists with different levels of experience, and between patients with different levels of sedation, these findings might serve as a valuable starting point for future studies.

Another issue to consider concerns the response bias that is inherent to questionnaire research. Patients with certain demographic characteristics or a specific colonoscopy experience (e.g. predominantly positive or negative) may be under- or overrepresented 37 . Comparison of both the responding and nonresponding patients would provide useful insights into the extent of the response bias. However, due to privacy regulations and the retrospective identification of patients through returned consent forms, identification of non-responders was not possible in this study. For future studies, a prospective patient counseling and consent procedure should be considered to (partially) address this issue.

As we adhered to a 30-day inclusion cutoff, this may have induced some degree of recall bias. However, the impact of recall bias appears limited as the rates of moderate-to-severe discomfort and pain were comparable between patients completing the questionnaire within or beyond the requested 2-day time frame. For future studies, a cutoff shorter than 30 days seems feasible given the high questionnaire completion rates within shorter time frames in our study (2 days 81%, 1 week 93%). Furthermore, all questionnaires were completed after the colonoscopy procedure. As some of the questionnaire sections cover the pre-procedural experience and patient’s expectations, patient responses could have been biased by the actual colonoscopy experience. Therefore, testing the questionnaire in two phases (before and after the procedure) would be useful for future studies. Partial completion of PREMs before the procedure may also facilitate useful insights into the patient’s current colonoscopy, rather than future colonoscopies only. Finally, as this study only involved two Dutch centers, the generalizability of our results may be compromised by factors such as standard sedation practices, patient population, and experience of the participating medical staff.

In conclusion, this study showed that clinician-reported GCS scores, used to indicate patient comfort during colonoscopy, frequently underestimated the level of discomfort and pain reported by patients themselves. The use of a validated PREM allowed for obtaining extensive insights into patients’ colonoscopy experiences from their perspectives and the identification of patient factors that might be associated with greater patient-reported discomfort and pain during colonoscopy. For these reasons, the use of validated PREMs could allow for more accurate monitoring of patients’ colonoscopy experiences compared with using the GCS as a standard measure for reporting patient comfort during colonoscopy.

## Supplementary Material

Supplementary Material Zusatzmaterial
