# Supplementary material for: Clinician-reported Gloucester Comfort Scale scores underestimate patient discomfort and pain during colonoscopy: insights from comparison with a patient-reported experience measure
Source: Endoscopy. 2025 Feb 28;57(6):645–57. doi: 10.1055/a-2528-5578 (PMC12132106; doi:10.1055/a-2528-5578)
Supplement: Supplementary file 1 — Supplementary Material [file 10-1055-a-2528-5578_25458355.pdf]

Clinician-reported Gloucester Comfort Scale scores underestimate patient discomfort and pain during colonoscopy: insights from comparison with a patient-reported experience measure

Querijn N. E. van Bokhorst, Charmayne V. Geerlings, Manon van der Vlugt, Karlijn J. Nass, Jos W. Borkent, Laura J. Neilson, Paul Fockens, Colin J. Rees, Evelien Dekker

Table 1s Overview of questions used to determine patient experience regarding specific domains of the colonoscopy and criteria for inclusion within the 'agree' group as described in Tables 6 & 7

Experience domain	Question(s)	Inclusion to 'agree' group in Tables 6 & 7 in case one of the questions is answered as underneath*
**Inadequate information**	B5: Before coming for the test, I was given enough information about what the test would involve<br>B6: After reading the information, I did not have any questions about the test<br>B8: I had enough time to discuss the test with the person who referred me<br>D3: I felt able to ask the staff any questions before the test<br>D4: I had no unanswered questions before the test	Disagree<br>Disagree<br>Disagree<br>Disagree<br>Disagree
**Anxiety: procedure in general**	B9: I felt anxious about what the test would involve<br>B10: I was made anxious by talking to other people who had previously had the test	Agree<br>Agree
**Anxiety: procedure results**	B11: I felt anxious about the results of the test	Agree
**Anxiety: procedure-related discomfort**	B12: I expected to experience discomfort during the test	Agree
**Anxiety: procedure-related pain**	B13: I expected to experience pain during the test<br>B14: I was worried that inserting the tube / camera would cause discomfort	Agree<br>Agree
**Bad experience bowel preparation**	C1: The preparation had an unpleasant taste<br>C2: The preparation tasted better than I had expected<br>AND<br>C3: The volume (*amount*) of the bowel preparation was more than I had expected<br>C4: The amount of bowel preparation I had to drink was manageable	Agree<br>Disagree<br><br>Agree<br>Disagree
**Unsatisfactory waiting times**	B2: The time from first being referred to having the test done was satisfactory<br>D1: The length of time I waited in the department was acceptable	Disagree<br>Disagree
**Insufficient privacy or unrespected dignity**	C6: I had enough privacy when getting ready for the test (*e.g. when changing clothes*)<br>D5: I had enough privacy when waiting for the test<br>D6: I had enough privacy when moving from the waiting area to the procedure room<br>E1: During the test my dignity was maintained at all times	Disagree<br>Disagree<br>Disagree<br>Disagree
**Endoscopist with unpreferred gender**	E4: I would have preferred the person doing the test (*inserting the tube or camera*) to be:<br>E5: The person doing the test was:	N/A†<br>N/A†
**Low sense of comfort and support (from the medical staff)**	D2: I was comfortable while sitting in the waiting area<br>E6: I felt confident that the person doing the test knew what they were doing<br>E7: The person doing the test did their best to put me at ease<br>E8: The other staff in the test room did their best to put me at ease<br>E9: I was satisfied with the explanation given to my about the test<br>E10: The person doing the test addressed any concerns I had<br>E11: I felt I could stop the test if it became too uncomfortable	Disagree<br>Disagree<br>Disagree<br>Disagree<br>Disagree<br>Disagree<br>Disagree
**Feelings of embarrassment**	E12: I felt embarrassed during the test	Agree
**Longer procedure duration than expected**	E13: The test took longer than expected	Agree

N/A, not available. *A patient was considered to 'disagree' in case the corresponding question was answered with either 'disagree' or 'strongly disagree', while patients were considered to 'agree' if the corresponding question was answered with 'agree' or 'strongly agree'; †Patients were assigned to the 'disagree' group in case the patient's preferred endoscopist gender matched the gender of the endoscopist that performed the procedure, or if patients indicated not to have a preference regarding the endoscopist's gender. Patients were assigned to the 'agree' group in case the patient's preferred endoscopist gender did not match the gender of the endoscopist that performed the procedure.

Table 2s Definition of different colonoscopy indication categories and number of included patients per indication

**CRCSP (n = 82)**	**Surveillance (n = 68)**	**Familial risk (n = 30)**	**Symptoms (n = 59)**	**Other (n = 4)**
Initial colonoscopy after positive FIT within the context of the Dutch CRCSP (n = 82)	Surveillance – after adenoma(s) (n = 34)	Familial risk – polyposis coli (n = 4)	Changed stool patterns (n = 13)	Abnormality found at imaging or peri-anal examination (n = 1)
	Surveillance – after CRC (n = 10)	Familial risk – CRC (n = 12)	Rectal blood loss (n = 13)	Positive FIT outside context of the Dutch CRCSP (n = 1)
	Surveillance – IBD (n = 21)	Familial risk – HNPCC / Lynch syndrome (n = 12)	Analysis iron deficiency anemia (n = 9)	Therapeutic colonoscopy (n = 1)
	Surveillance – after excision colorectal lesion (n = 3)	Familial risk – other (n = 2)	Chronic diarrhea (n = 5)	Evaluation of (endoscopic) treatment possibilities of colorectal lesions (n = 1)
			Abdominal pain (n = 12)	
			Suspicion of IBD (n = 3)	
			Evaluation of disease activity of IBD (n = 4)	

CRCSP, colorectal cancer screening programme; FIT, faecal immunochemical test; CRC, colorectal cancer; IBD, inflammatory bowel disease; HNPCC, hereditary non-polyposis colorectal cancer.

Table 3s Matching percentages and levels of agreement between clinician- and patient-reported scores for specific subgroups

		Discomfort				Pain			
		Match, n (%)	GCS lower, n (%)	GCS higher, n (%)	Cohen's κ (95% CI)*	Match, n (%)	GCS lower, n (%)	GCS higher, n (%)	Cohen's κ (95% CI)*
**Type of assessor**	Nurse (n = 182)	98 (54)	42 (23)	42 (23)	0.34 (0.15-0.49)	105 (58)	44 (24)	33 (18)	0.52 (0.38-0.65)
	Endoscopist (n = 61)	21 (34)	30 (49)	10 (16)	0.32 (0.10-0.54)	28 (46)	27 (44)	6 (9.8)	0.33 (0.15-0.52)
**Experience level of endoscopist**	Gastroenterologist (n = 182)	91 (50)	48 (26)	43 (24)	0.27 (0.11-0.44)	98 (54)	50 (27)	34 (19)	0.40 (0.26-0.53)
	Gastroenterologist in training (n = 61)	28 (46)	24 (39)	9 (15)	0.47 (0.25-0.64)	35 (57)	21 (34)	5 (8.2)	0.61 (0.43-0.75)
**Degree of sedation†**	Leeds score 1 (n = 116)	68 (59)	30 (26)	18 (16)	0.20 (0.01-0.37)	74 (64)	29 (25)	13 (11)	0.38 (0.21-0.55)
	Leeds score ≥2 (n = 125)	50 (40)	41 (33)	34 (27)	0.33 (0.15-0.50)	57 (46)	42 (34)	26 (21)	0.45 (0.28-0.59)

*****The Cohen's κ statistic represents the level of agreement between the clinician reported GCS-scores and patient-reported scores. Reported Cohen's κ values can be interpreted according to the recommendations by McHugh (2012): 0-0.20: none, 0.21-0.39: minimal, 0.40-0.59: weak, 0.60-0.79: moderate, 0.80-0.90: strong, >0.90: almost perfect.; †Two patients for whom no Leeds score was reported were excluded from the analyses.

Table 4s. Frequency of specific score counts for clinician-reported GCS scores and patient-reported discomfort and pain scores in relation to (dose of) administered medication

Medication type	Medication		Number of patients, n (%)	Score	Score count, n				
	Dose midazolam (mg)	Dose fentanyl (mg)			1	2	3	4	5
**None**	None	None	**28 (12)**	GCS	22	6	0	0	0
				Discomfort	15	8	3	2	0
				Pain	16	7	4	1	0
**Midazolam only**	<2.5	None	**0**	GCS	0	0	0	0	0
				Discomfort	0	0	0	0	0
				Pain	0	0	0	0	0
	2.5	None	**1 (0.4)**	GCS	1	0	0	0	0
				Discomfort	1	0	0	0	0
				Pain	1	0	0	0	0
	>2.5	None	**1 (0.4)**	GCS	1	0	0	0	0
				Discomfort	0	1	0	0	0
				Pain	0	1	0	0	0
**Fentanyl only**	None	<0.05	**0**	GCS	0	0	0	0	0
				Discomfort	0	0	0	0	0
				Pain	0	0	0	0	0
	None	0.05	**4 (1.6)**	GCS	4	0	0	0	0
				Discomfort	2	2	0	0	0
				Pain	2	1	1	0	0
	None	>0.05	**4 (1.6)**	GCS	3	1	0	0	0
				Discomfort	3	1	0	0	0
				Pain	3	1	0	0	0
**Midazolam and fentanyl**	<2.5	<0.05	**1 (0.4)**	GCS	1	0	0	0	0
				Discomfort	1	0	0	0	0
				Pain	1	0	0	0	0
	<2.5	0.05	**3 (1.2)**	GCS	2	0	1	0	0
				Discomfort	1	0	0	2	0
				Pain	1	1	1	0	0
	<2.5	>0.05	**1 (0.4)**	GCS	1	0	0	0	0
				Discomfort	1	0	0	0	0
				Pain	1	0	0	0	0
	2.5	<0.05	**4 (1.6)**	GCS	2	2	0	0	0
				Discomfort	2	1	1	0	0
				Pain	1	3	0	0	0
	2.5	0.05	**142 (58)**	GCS	91	49	2	0	0
				Discomfort	93	23	15	10	1
				Pain	98	18	13	12	1
	2.5	>0.05	**20 (8.2)**	GCS	0	13	7	0	0
				Discomfort	9	4	1	5	1
				Pain	4	2	4	9	1
	>2.5	<0.05	**0**	GCS	0	0	0	0	0
				Discomfort	0	0	0	0	0
				Pain	0	0	0	0	0
	>2.5	0.05	**22 (9.1)**	GCS	8	12	1	1	0
				Discomfort	14	2	3	3	0
				Pain	12	4	3	3	0
	>2.5	>0.05	**12 (4.9)**	GCS	1	5	3	2	1
				Discomfort	4	2	0	4	2
				Pain	2	3	1	4	2

GCS, Gloucester Comfort Scale. Notes: all patients with a GCS $\geq$3 (n = 18) received medication. Compared to standard doses of fentanyl and midazolam, 15/18 (83%) of these patients received either extra fentanyl (n = 7), midazolam (n = 2) or both (n = 6). Of patients that reported a discomfort score $\geq$3, 48/53 (91%) received medication. The majority of these patients (n = 45) received at least a standard dose of both fentanyl and midazolam, while 19/53 (36%) patients received a higher than standard dose of fentanyl (n = 7), midazolam (n = 6) or both (n = 6). Of patients that reported a pain score $\geq$3, 55/60 (92%) received medication. A higher than standard dose of fentanyl (n = 14), midazolam (n = 6) or both (n = 6) was administered to 26/60 (43%) patients.

Table 5s Univariable and multivariable regression analyses assessing the association between different patient- and procedural factors and moderate to severe patient-reported discomfort (extended table)

				Univariable analysis		Multivariable analysis*	
	**Variable**	**No to mild discomfort, n (%)**	**Moderate to severe discomfort, n (%)**	**OR (95% CI)**	**P-value**	**OR (95% CI)**	**P-value**
**Sex**	Male	102 (86)	16 (14)	Reference		Reference	
	Female	88 (70)	37 (30)	2.68 (1.40-5.15)	0.003	2.46 (1.25-4.86)	0.009
**Age**	≥55	169 (83)	35 (17)	Reference		Reference	
	<55	21 (54)	18 (46)	4.14 (2.00-8.56)	<0.001	2.91 (1.33-6.36)	0.007
**Educational level†**	Low	43 (83)	9 (17)	Reference		Reference	
	Medium or high	138 (77)	42 (23)	1.45 (0.66-3.23)	0.357	1.12 (0.48-2.59)	0.797
**Body mass index‡**	18.5-25.0	89 (75)	29 (25)	Reference		Reference	
	>25.0	100 (81)	24 (19)	0.74 (0.40-1.36)	0.327	0.98 (0.51-1.90)	0.959
**Previous abdominal surgery**	No	147 (80)	37 (20)	Reference		Reference	
	Yes	43 (73)	16 (27)	1.48 (0.75-2.91)	0.258	1.40 (0.68-2.89)	0.357
**Previous colonoscopy**	No	92 (82)	20 (18)	Reference		Reference	
	Yes	98 (75)	33 (25)	1.55 (0.83-2.89)	0.169	1.16 (0.57-2.37)	0.681
**Diverticulosis sigmoid**	No	108 (77)	32 (23)	Reference		Reference	
	Yes	82 (80)	21 (20)	0.86 (0.46-1.61)	0.644	1.64 (0.79-3.41)	0.187
**Colonoscopy indication**	CRCSP	76 (93)	6 (7)	Reference		Reference	
	Surveillance and familial risk	70 (71)	28 (29)	5.07 (1.98-12.97)	<0.001	3.22 (1.13-9.21)	0.029
	Symptoms and other	44 (70)	19 (30)	5.47 (2.03-14.72)	<0.001	3.77 (1.33-10.70)	0.013
**Endoscopist experience**	Gastroenterologist	148 (81)	34 (19)	Reference		Reference	
	Gastroenterologist in training	42 (69)	19 (31)	1.97 (1.02-3.80)	0.043	1.60 (0.77-3.32)	0.158
**Endoscopist type¶**	CRCSP accredited	118 (80)	30 (20)	Reference		Reference	
	Not CRCSP accredited	30 (88)	4 (12)	0.52 (0.17-1.60)	0.258	0.41 (0.12-1.40)	0.156

OR, odds ratio; CI, confidence interval; CRCSP, Colorectal Cancer Screening Programme. *Adjusted for gender, age (dichotomised at 55 years) and endoscopy centre; †Education level according to ISCED-11. Patients were considered to have an intermediate or high educational level if they had at least an upper secondary or university degree; ‡One patient with BMI <18.5 was excluded from the analyses; ¶To assure colonoscopy quality for colonoscopies performed within the context of the Dutch CRCSP, all endoscopists performing these procedures have to be accredited. The endoscopist accreditation programme consists of three modules: (1) colonoscopy registration module, (2) theoretical e-learning module combined with online assessment of the acquired knowledge and (3) a practical evaluation of colonoscopy and polypectomy skills.

Table 6s Univariable and multivariable regression analyses assessing the association between different patient- and procedural factors and moderate to severe patient-reported pain (extended table)

				Univariable analysis		Multivariable analysis*	
	**Variable**	**No to mild discomfort, n (%)**	**Moderate to severe discomfort, n (%)**	OR (95% CI)	P-value	OR (95% CI)	P-value
**Sex**	Male	100 (85)	18 (15)	Reference			
	Female	83 (66)	42 (34)	2.81 (1.51-5.23)	<0.001	2.68 (1.41-5.09)	0.003
**Age**	≥55	161 (79)	43 (21)	Reference			
	<55	22 (56)	17 (44)	2.89 (1.41-5.92)	0.004	2.01 (0.92-4.36)	0.078
**Educational level†**	Low	40 (77)	12 (23)	Reference			
	Medium or high	133 (74)	47 (26)	1.18 (0.57-2.43)	0.656	0.99 (0.46-2.12)	0.972
**Body mass index‡**	18.5-25.0	85 (72)	33 (28)	Reference			
	<18.5 or >25.0	98 (78)	27 (22)	0.71 (0.40-1.27)	0.250	0.86 (0.46-1.60)	0.628
**Previous abdominal surgery**	No	142 (77)	42 (23)	Reference			
	Yes	41 (69)	18 (31)	1.48 (0.77-2.85)	0.241	1.37 (0.69-2.72)	0.369
**Previous colonoscopy**	No	86 (77)	26 (23)	Reference			
	Yes	97 (74)	34 (26)	1.16 (0.64-2.09)	0.621	0.85 (0.43-1.67)	0.636
**Diverticulosis sigmoid**	No	109 (78)	31 (22)	Reference			
	Yes	74 (72)	29 (28)	1.38 (0.77-2.48)	0.248	2.26 (1.11-4.58)	0.024
**Colonoscopy indication**	CRCSP	69 (84)	13 (16)	Reference			
	Surveillance and familial risk	72 (73)	26 (27)	1.92 (0.31-4.03)	0.086	1.06 (0.43-2.61)	0.893
	Symptoms and other	42 (67)	21 (33)	2.65 (1.20-5.85)	0.016	1.82 (0.78-4.29)	0.169
**Endoscopist experience**	Gastroenterologist	140 (77)	42 (23)	Reference			
	Gastroenterologist in training	43 (70)	18 (30)	1.40 (0.73-2.67)	0.320	1.10 (0.54-2.26)	0.787
**Endoscopist type¶**	CRCSP accredited	113 (76)	35 (24)	Reference			
	Not CRCSP accredited	70 (74)	25 (26)	1.15 (0.64-2.09)	0.639	0.81 (0.31-2.11)	0.664

OR, odds ratio; CI, confidence interval; CRCSP, Colorectal Cancer Screening Programme. *Adjusted for gender, age (dichotomised at 55 years) and endoscopy centre; †Education level according to ISCED-11. Patients were considered to have an intermediate or high educational level if they had at least an upper secondary or university degree; ‡One patient with BMI <18.5 was excluded from the analyses; ¶To assure colonoscopy quality for colonoscopies performed within the context of the Dutch CRCSP, all endoscopists performing these procedures have to be accredited. The endoscopist accreditation programme consists of three modules: (1) colonoscopy registration module, (2) theoretical e-learning module combined with online assessment of the acquired knowledge and (3) a practical evaluation of colonoscopy and polypectomy skills.

Table 7s. Univariable regression analyses assessing the association between various patient- and procedure-related factors and over- and underestimation of patient-reported discomfort using the GCS

		Overestimation				Underestimation			
	**Variable**	**Match, n (%)**	**GCS score higher, n (%)**	**OR (95% CI)**	**P-value**	**Match, n (%)**	**GCS score lower, n (%)**	**OR (95% CI)**	**P-value**
**Sex**	Male	67 (74)	23 (26)	Reference		67 (71)	28 (29)	Reference	
	Female	52 (64)	29 (36)	1.62 (0.84-3.13)	0.147	52 (54)	44 (46)	2.02 (1.12-3.68)	0.020
**Age**	≥55	103 (68)	48 (32)	Reference		103 (66)	53 (34)	Reference	
	<55	16 (80)	4 (20)	0.54 (0.17-1.69)	0.288	16 (46)	19 (54)	2.31 (1.10-4.85)	0.027
**Educational level***	Low	28 (65)	15 (35)	Reference		28 (76)	9 (24)	Reference	
	Medium or high	86 (72)	34 (28)	0.74 (0.35-1.55)	0.422	86 (59)	60 (41)	2.17 (0.96-4.93)	0.064
**Body mass index†**	18.5-25.0	54 (68)	25 (32)	Reference		54 (58)	39 (42)	Reference	
	>25.0	65 (71)	26 (29)	0.86 (0.45-1.67)	0.663	65 (66)	33 (34)	0.70 (0.39-1.26)	0.240
**Previous abdominal surgery**	No	89 (70)	38 (30)	Reference		89 (61)	57 (39)	Reference	
	Yes	30 (68)	14 (32)	1.09 (0.52-2.29)	0.814	30 (67)	15 (33)	0.78 (0.39-1.58)	0.490
**Previous colonoscopy**	No	61 (70)	26 (30)	Reference		61 (71)	25 (29)	Reference	
	Yes	58 (69)	26 (31)	1.05 (0.55-2.02)	0.879	58 (55)	47 (45)	1.98 (1.08-3.62)	0.027
**Diverticulosis sigmoid**	No	69 (73)	25 (27)	Reference		69 (60)	46 (40)	Reference	
	Yes	50 (65)	27 (35)	1.49 (0.77-2.87)	0.232	50 (66)	26 (34)	0.78 (0.43-1.43)	0.419
**Colonoscopy indication**	CRCSP	50 (72)	19 (28)	Reference		50 (79)	13 (21)	Reference	
	Surveillance and familial risk	40 (69)	18 (31)	1.18 (0.55-2.55)	0.666	40 (50)	40 (50)	3.85 (1.81-8.15)	<0.001
	Symptoms and other	29 (66)	15 (34)	1.36 (0.60-3.08)	0.460	29 (60)	19 (40)	2.52 (1.09-5.84)	0.031
**Endoscopist experience**	Gastroenterologist	91 (68)	43 (32)	Reference		91 (65)	48 (35)	Reference	
	Gastroenterologist in training	28 (76)	9 (24)	0.68 (0.30-1.57)	0.365	28 (54)	24 (46)	1.62 (0.85-3.11)	0.142
**Endoscopist type‡**	CRCSP accredited	72 (69)	33 (31)	Reference		72 (63)	43 (37)	Reference	
	Not CRCSP accredited	19 (66)	10 (34)	1.15 (0.48-2.74)	0.755	19 (79)	5 (21)	0.44 (0.15-1.27)	0.108

GCS, Gloucester Comfort Scale; OR, odds ratio; CI, confidence interval; CRCSP, Colorectal Cancer Screening Programme. *Education level according to ISCED-11. Patients were considered to have an intermediate or high educational level if they had at least an upper secondary or university degree; †One patient with BMI <18.5 was excluded from the analyses; ‡To assure colonoscopy quality for colonoscopies performed within the context of the Dutch CRCSP, all endoscopists performing these procedures have to be accredited. The endoscopist accreditation programme consisting of three modules: (1) colonoscopy registration module, (2) theoretical e-learning module combined with online assessment of the acquired knowledge and (3) a practical evaluation of colonoscopy and polypectomy skills.

Table 8s Univariable regression for assessment of the association between various patient- and procedure-related factors and over- and underestimation of patient-reported pain using the GCS

		Overestimation				Underestimation			
	**Variable**	**Match, n (%)**	**GCS score higher, n (%)**	**OR (95% CI)**	**P-value**	**Match, n (%)**	**GCS score lower, n (%)**	**OR (95% CI)**	**P-value**
**Sex**	Male	100 (85)	18 (15)	Reference		77 (75)	26 (25)	Reference	
	Female	83 (66)	42 (34)	2.81 (1.51-5.25)	0.001	56 (55)	45 (45)	2.38 (1.32-4.31)	0.004
**Age**	≥55	161 (79)	43 (21)	Reference		116 (69)	52 (31)	Reference	
	<55	22 (56)	17 (44)	2.89 (1.41-5.92)	0.004	17 (47)	19 (53)	2.49 (1.20-5.18)	0.014
**Educational level***	Low	40 (77)	12 (23)	Reference		28 (72)	11 (28)	Reference	
	Medium or high	133 (74)	47 (26)	1.18 (0.57-2.43)	0.656	98 (63)	57 (37)	1.48 (0.69-3.20)	0.318
**Body mass index†**	18.5-25.0	85 (72)	33 (28)	Reference		61 (60)	40 (40)	Reference	
	>25.0	98 (79)	26 (21)	0.68 (0.38-1.23)	0.206	72 (71)	30 (29)	0.64 (0.35-1.14)	0.128
**Previous abdominal surgery**	No	142 (77)	42 (23)	Reference		104 (66)	53 (34)	Reference	
	Yes	41 (69)	18 (31)	1.48 (0.77-2.85)	0.235	29 (62)	18 (38)	1.22 (0.62-2.39)	0.567
**Previous colonoscopy**	No	86 (77)	26 (23)	Reference		65 (71)	27 (29)	Reference	
	Yes	97 (74)	34 (26)	1.16 (0.64-2.09)	0.622	68 (61)	44 (39)	1.56 (0.87-2.80)	0.137
**Diverticulosis sigmoid**	No	82 (81)	19 (19)	Reference		82 (68)	39 (32)	Reference	
	Yes	71 (72)	20 (28)	1.69 (0.82-3.47)	0.151	51 (61)	32 (39)	1.32 (0.74-2.36)	0.352
**Colonoscopy indication**	CRCSP	76 (93)	6 (7)	Reference		55 (80)	14 (20)	Reference	
	Surveillance and familial risk	70 (71)	28 (29)	5.07 (1.98-12.97)	≤0.001	51 (59)	35 (41)	2.70 (1.30-5.58)	0.008
	Symptoms and other	44 (70)	19 (30)	5.47 (2.03-14.72)	≤0.001	27 (55)	22 (45)	3.20 (1.42-7.22)	0.005
**Endoscopist experience**	Gastroenterologist	140 (77)	42 (23)	Reference		98 (66)	50 (34)	Reference	
	Gastroenterologist in training	43 (70)	18 (30)	1.40 (0.73-2.67)	0.320	35 (63)	21 (38)	1.18 (0.62-2.23)	0.620
**CRCSP certification**	CRCSP certified	113 (76)	35 (24)	Reference		83 (67)	4	Reference	
							1 (33)		
	Not CRCSP certified	27 (79)	7 (21)	0.84 (0.34-2.09)	0.703	15 (63)	9 (38)	1.21 (0.49-3.01)	0.676

GCS, Gloucester Comfort Scale; OR, odds ratio; CI, confidence interval; CRCSP, Colorectal Cancer Screening Programme. *Education level according to ISCED-11. Patients were considered to have an intermediate or high educational level if they had at least an upper secondary or university degree; †One patient with BMI <18.5 was excluded from the analyses; ‡To assure colonoscopy quality for colonoscopies performed within the context of the Dutch CRCSP, all

endoscopists performing these procedures have to be accredited. The endoscopist accreditation programme consisting of three modules: (1) colonoscopy registration module, (2) theoretical e-learning module combined with online assessment of the acquired knowledge and (3) a practical evaluation of colonoscopy and polypectomy skills.

Fig. 1s Plot illustrating the agreement between patient-reported discomfort and pain scores. The number of patients that is represented by each data point within the plot is indicated by the size of each datapoint and the number within each datapoint. The Goodman and Kruskal's gamma reports the strength and direction of the association between patient-reported scores and indicates a strong positive association between discomfort and pain scores (Goodman and Kruskal's γ: 0.78 [95% CI: 0.69-0.86]).

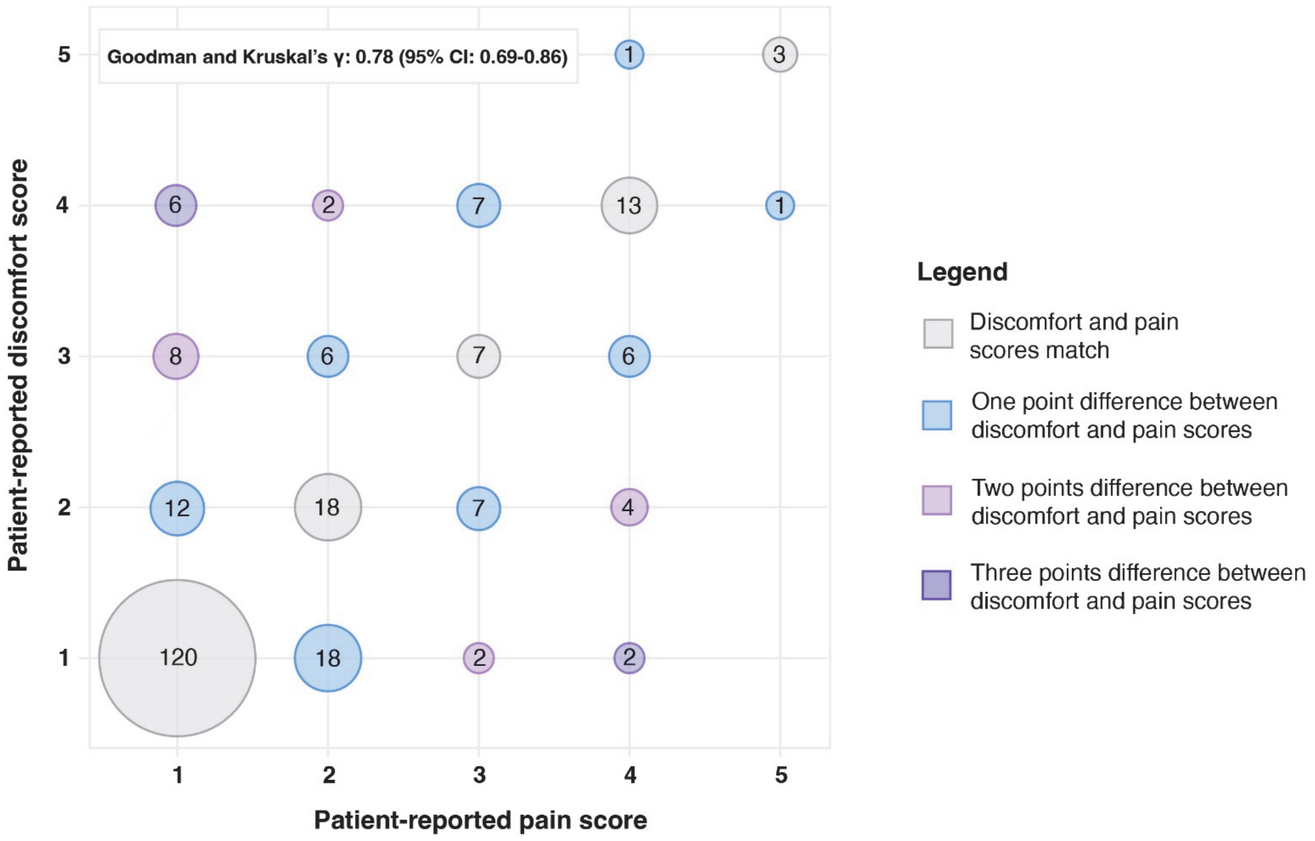

Appendix 1s Overview of questions included within the Newcastle ENDOPREM colonoscopy questionnaire, adapted for the Dutch population (English version)

Note: development and validation of this questionnaire will be described elsewhere.

SECTION A: Completing this survey

A1. Please fill in today's date
d: _____ m: _____ y: _____

A2. How long ago was your most recent test?
Weeks: _____ Days: ______

A3. Please fill in your age (in years)
_____

A4. Are you?
❍ Male ❍ Female ❍ Other ❍ Prefer not to tell

A5. How many years of full time education have you completed (*starting from age 5*)?
_____

A6. To which of these ethnic groups would you say you belong?
- ❍ West-European (*including the Netherlands, Germany or any other West-European country*)
- ❍ East-European (*including Poland, Russia or any other East-European country)*
- ❍ Asian (*including Indonesia, India or any other Asian country*)
- ❍ Middle East (*including Syria, Iraq, Afghanistan or any other country within the Middle East*)
- ❍ Mediterranean (*including Turkey, Morocco or any other mediterranean country)*
- ❍ Caribbean and Surinam (*including (former) Netherlands Antilles and Surinam)*

A7. Please tell us if someone is helping you complete this survey
- ❍ I am completing this survey by myself
- ❍ Someone is helping me complete the survey

A8. Have you ever had another camera test (endoscopy) of the stomach or large bowel or a CT scan (CT colonography) of the large bowel?
❍ Yes ❍ No

Excluding your most recent rest, please indicate which test and how many you have had
- ❍ Colonoscopy (*camera or tube inserted though the back passage*) Number: _____
- ❍ Gastroscopy (*camera or tube inserted though the mouth into the stomach*) Number: _____
- ❍ Transnasal gastroscopy (*camera or tube inserted through the nose into the stomach*) Number: _____
- ❍ CT colonography (*CT scan where a short tube is inserted into the back passage – done in the X-ray department)* Number: _____
- ❍ Flexible sigmoidoscopy (*camera inserted through the back passage into the last part of the bowel only – usually only requires an enema*) Number: _____

A9. How were you referred for your most recent test?
- ❍ I was referred directly by my general practitioner (without seeing a hospital doctor)
- ❍ The test was organised by a hospital doctor
- ❍ I have regular test to monitor a medical condition / because of my family history
- ❍ I was referred through the national bowel cancer screening programme
- ❍ I was referred another way (please tell us more below)

____

SECTION B: Before coming to the hospital for your test

B1. I was happy with the way I was referred for the test
❍ Strongly agree ❍ Agree ❍ Neither agree or disagree ❍ Disagree ❍ Strongly disagree

B2. The time from first being referred to having the test done was satisfactory
❍ Strongly agree ❍ Agree ❍ Neither agree or disagree ❍ Disagree ❍ Strongly disagree

B3. I felt able to change the appointment if it didn't suit me
❍ Strongly agree ❍ Agree ❍ Neither agree or disagree ❍ Disagree ❍ Strongly disagree

B4. My appointment was cancelled or changed by the hospital
❍ Yes ❍ No ❍ Not sure / cannot remember

B5. Before coming for the test, I was given enough information about what the test would involve
❍ Strongly agree ❍ Agree ❍ Neither agree or disagree ❍ Disagree ❍ Strongly disagree

B6. After reading the information, I did not have any questions about the test
❍ Strongly agree ❍ Agree ❍ Neither agree or disagree ❍ Disagree ❍ Strongly disagree

B7. The instructions on what I needed to do before the test were easy to follow
❍ Strongly agree ❍ Agree ❍ Neither agree or disagree ❍ Disagree ❍ Strongly disagree

B8. I had enough time to discuss the test with the person who referred me
❍ Strongly agree ❍ Agree ❍ Neither agree or disagree ❍ Disagree ❍ Strongly disagree

B9. I felt anxious about what the test would involve
❍ Strongly agree ❍ Agree ❍ Neither agree or disagree ❍ Disagree ❍ Strongly disagree

B10. I was made anxious by talking to other people who had previously had the test
❍ Strongly agree ❍ Agree ❍ Neither agree or disagree ❍ Disagree ❍ Strongly disagree

B11. I felt anxious about the results of the test
❍ Strongly agree ❍ Agree ❍ Neither agree or disagree ❍ Disagree ❍ Strongly disagree

B12. I expected to experience discomfort during the test
❍ Strongly agree ❍ Agree ❍ Neither agree or disagree ❍ Disagree ❍ Strongly disagree

B13. I expected to experience pain during the test
❍ Strongly agree ❍ Agree ❍ Neither agree or disagree ❍ Disagree ❍ Strongly disagree

B14. I was worried that inserting the tube/camera would cause discomfort
❍ Strongly agree ❍ Agree ❍ Neither agree or disagree ❍ Disagree ❍ Strongly disagree

SECTION C: Preparing for you test

C1. The bowel preparation had an unpleasant taste
❍ Strongly agree ❍ Agree ❍ Neither agree or disagree ❍ Disagree ❍ Strongly disagree

C2. The bowel preparation tasted better than I had expected
❍ Strongly agree ❍ Agree ❍ Neither agree or disagree ❍ Disagree ❍ Strongly disagree

C3. The volume (*amount*) of the bowel preparation was more than I had expected
❍ Strongly agree ❍ Agree ❍ Neither agree or disagree ❍ Disagree ❍ Strongly disagree

C4. The amount of bowel preparation I had to drink was manageable
❍ Strongly agree ❍ Agree ❍ Neither agree or disagree ❍ Disagree ❍ Strongly disagree

C5. I was worried that the bowel preparation would not clear my bowel properly
❍ Strongly agree ❍ Agree ❍ Neither agree or disagree ❍ Disagree ❍ Strongly disagree

C6. I had enough privacy when getting ready for the test (*e.g., when changing clothes)*
❍ Strongly agree ❍ Agree ❍ Neither agree or disagree ❍ Disagree ❍ Strongly disagree

SECTION D: At the hospital, before the test

D1. The length of time I waited in the department was acceptable
❍ Strongly agree ❍ Agree ❍ Neither agree or disagree ❍ Disagree ❍ Strongly disagree

D2. I was comfortable while sitting in the waiting area
❍ Strongly agree ❍ Agree ❍ Neither agree or disagree ❍ Disagree ❍ Strongly disagree

D3. I felt able to ask the staff any questions before the test
❍ Strongly agree ❍ Agree ❍ Neither agree or disagree ❍ Disagree ❍ Strongly disagree

D4. I had no unanswered questions before the test
❍ Strongly agree ❍ Agree ❍ Neither agree or disagree ❍ Disagree ❍ Strongly disagree

D5. I had enough privacy when waiting for the test
❍ Strongly agree ❍ Agree ❍ Neither agree or disagree ❍ Disagree ❍ Strongly disagree

D6. I had enough privacy when moving from the waiting area to the procedure room
❍ Strongly agree ❍ Agree ❍ Neither agree or disagree ❍ Disagree ❍ Strongly disagree

SECTION E: During the test

E1. During the test my dignity was maintained at all times
❍ Strongly agree ❍ Agree ❍ Neither agree or disagree ❍ Disagree ❍ Strongly disagree

E2. I felt free to choose what medication to take (*e.g., sedative, no medication*)
❍ Strongly agree ❍ Agree ❍ Neither agree or disagree ❍ Disagree ❍ Strongly disagree

E3. The medication worked as well as I had expected
❍ Strongly agree ❍ Agree ❍ Neither agree or disagree ❍ Disagree ❍ Strongly disagree
❍ I didn't have any medication

E4. I would have preferred the person doing the test (*inserting the tube or camera*) to be:
❍ Male ❍ Female ❍ I have no preference

E5. The person doing the test was:
❍ Male ❍ Female

E6. I felt confident that the person doing the test knew what they were doing
❍ Strongly agree ❍ Agree ❍ Neither agree or disagree ❍ Disagree ❍ Strongly disagree

E7. The person doing the test did their best to put me at ease
❍ Strongly agree ❍ Agree ❍ Neither agree or disagree ❍ Disagree ❍ Strongly disagree

E8. The other staff in the test room did their best to put me at ease
❍ Strongly agree ❍ Agree ❍ Neither agree or disagree ❍ Disagree ❍ Strongly disagree

E9. I was satisfied with the explanation given to me about the test
❍ Strongly agree ❍ Agree ❍ Neither agree or disagree ❍ Disagree ❍ Strongly disagree

E10. The person doing the test addressed any concerns I had
❍ Strongly agree ❍ Agree ❍ Neither agree or disagree ❍ Disagree ❍ Strongly disagree

E11. I felt I could stop the test if it became too uncomfortable
❍ Strongly agree ❍ Agree ❍ Neither agree or disagree ❍ Disagree ❍ Strongly disagree
❍ *I slept during the procedure*

E12. I felt embarrassed during the test
❍ Strongly agree ❍ Agree ❍ Neither agree or disagree ❍ Disagree ❍ Strongly disagree
❍ *I slept during the procedure*

E13. The test took long than I expected
❍ Strongly agree ❍ Agree ❍ Neither agree or disagree ❍ Disagree ❍ Strongly disagree
❍ *I slept during the procedure*

E14. How would you rate the level of discomfort you experienced during the test? Please circle a number below:

No discomfort	0	1	2	3	4	5	6	7	8	9	10	Worst discomfort imaginable

❍ *I slept during the procedure*

E15. How long did the discomfort last during the test?
❍ I didn't have discomfort ❍ A short time ❍ A moderate time ❍ A long time
❍ *I slept during the procedure*

E16. How many times did you experience discomfort during the test?
❍ None ❍ 1 or 2 times ❍ 3 or 4 times ❍ More than 4 times ❍ Constantly
❍ *I slept during the procedure*

E17. How would you rate the level of pain you experienced during the test? Please circle a number below:

No pain	0	1	2	3	4	5	6	7	8	9	10	Worst pain imaginable

❍ *I slept during the procedure*

E18. How long did the pain last during the test?
❍ I didn't have pain ❍ A short time ❍ A moderate time ❍ A long time
❍ *I slept during the procedure*

E19. How many times did you experience pain during the test?
❍ None ❍ 1 or 2 times ❍ 3 or 4 times ❍ More than 4 times ❍ Constantly
❍ *I slept during the procedure*

E20. Overall, I experienced more discomfort than I expected during the test
❍ Strongly agree ❍ Agree ❍ Neither agree or disagree ❍ Disagree ❍ Strongly disagree
❍ *I slept during the procedure*

E21. Overall, I experience more pain than I expected during the test
❍ Strongly agree ❍ Agree ❍ Neither agree or disagree ❍ Disagree ❍ Strongly disagree
❍ *I slept during the procedure*

E22. I felt embarrassed by the discomfort I experienced
❍ Strongly agree ❍ Agree ❍ Neither agree or disagree ❍ Disagree ❍ Strongly disagree
❍ *I slept during the procedure*

E23. I felt embarrassed by the pain I experience
❍ Strongly agree ❍ Agree ❍ Neither agree or disagree ❍ Disagree ❍ Strongly disagree
❍ *I slept during the procedure*

SECTION F: After the test

F1. I was satisfied by the explanation given to me by the person doing the test
❍ Strongly agree ❍ Agree ❍ Neither agree or disagree ❍ Disagree ❍ Strongly disagree

F2. I had discomfort after the test
❍ Strongly agree ❍ Agree ❍ Neither agree or disagree ❍ Disagree ❍ Strongly disagree

F3. It took longer than I expected to recover from the test
❍ Strongly agree ❍ Agree ❍ Neither agree or disagree ❍ Disagree ❍ Strongly disagree

F4. I was worried about the test results
❍ Strongly agree ❍ Agree ❍ Neither agree or disagree ❍ Disagree ❍ Strongly disagree

F5. Have you received the results of your test (*please tick all that apply*)
❍ Yes, I have received all of my test results
❍ Yes, I have received some of my test results
❍ No

F6. When I left the hospital, I was clear about what the next steps would be
❍ Strongly agree ❍ Agree ❍ Neither agree or disagree ❍ Disagree ❍ Strongly disagree

F7. I was happy with the way I received the results of my test
❍ Strongly agree ❍ Agree ❍ Neither agree or disagree ❍ Disagree ❍ Strongly disagree
❍ *I do not have my results*

F8. I received the results of my test sooner than I had expected
❍ Strongly agree ❍ Agree ❍ Neither agree or disagree ❍ Disagree ❍ Strongly disagree
❍ *I do not have my results*

SECTION G: Overall experience

G1. Overall I was satisfied with my experience of the test
❍ Strongly agree ❍ Agree ❍ Neither agree or disagree ❍ Disagree ❍ Strongly disagree

G2. If there is something else you would like to tell us about your test, please use the space below

____
